# Correlates of high-impact physical activity measured objectively in older British adults

**DOI:** 10.1093/pubmed/fdx171

**Published:** 2017-12-11

**Authors:** Ahmed Elhakeem, Kimberly Hannam, Kevin C Deere, April Hartley, Emma M Clark, Charlotte Moss, Mark H Edwards, Elaine Dennison, Tim Gaysin, Diana Kuh, Andrew Wong, Kenneth R Fox, Cyrus Cooper, Rachel Cooper, Jon H Tobias

**Affiliations:** 1 Musculoskeletal Research Unit, Translational Health Sciences, Bristol Medical School, University of Bristol, Bristol, UK; 2 MRC Lifecourse Epidemiology Unit, University of Southampton, Southampton, UK; 3 MRC Unit for Lifelong Health and Ageing at UCL, London, UK; 4 Centre for Exercise Nutrition and Health Sciences, University of Bristol, Bristol, UK

**Keywords:** accelerometer, ageing, epidemiology, physical activity, vertical impacts

## Abstract

**Background:**

Exposure to higher magnitude vertical impacts is thought to benefit bone health. The correlates of this high-impact physical activity (PA) in later life are unknown.

**Methods:**

Participants were from the Cohort for Skeletal Health in Bristol and Avon, Hertfordshire Cohort Study and MRC National Survey of Health and Development. Associations of demographic, behavioural, physiological and psychological factors with vertical acceleration peaks ≥1.5 g (i.e. high-impact PA) from 7-day hip-worn accelerometer recordings were examined using linear regression.

**Results:**

A total of 1187 participants (mean age = 72.7 years, 66.6% females) were included. Age, sex, education, active transport, self-reported higher impact PA, walking speed and self-rated health were independently associated with high-impact PA whereas BMI and sleep quality showed borderline independent associations. For example, differences in log-high-impact counts were 0.50 (*P* < 0.001) for men versus women and −0.56 (*P* < 0.001) for worst versus best self-rated health. Our final model explained 23% of between-participant variance in high impacts. Other correlates were not associated with high-impact activity after adjustment.

**Conclusions:**

Besides age and sex, several factors were associated with higher impact PA in later life. Our findings help identify characteristics of older people that might benefit from interventions designed to promote osteogenic PA.

## Background

The progressive age-related disorder of osteoporosis, characterized by loss of bone mass and strength leading to fragility fractures, has large associated societal costs that are expected to rise for future generations.^1^ Physical activity (PA) produces wide ranging benefits for older adults that include increases in bone mineral density,^2^ prevention of falls and fractures and the maintenance of independent living^3–5^ and physical capability.^6,7^ Importantly, it is thought that beneficial effects of PA on bone are mediated by deformations caused by higher impacts or loading forces, leading to new bone growth, which subsequently reduces risk of osteoporosis.^8–11^ For example, we recently developed^12^ and validated^13^ an accelerometer-based method for characterizing PA according to vertical impact, and showed that positive associations with lower limb bone strength in postmenopausal women were explained by exposure to vertical impacts ≥1.5 g.^8^ To underpin strategies to increase older adults’ exposure to higher impact PA, greater understanding is needed of the determinants of high-impact PA.

Previous studies examining predictors of older people’s PA have primarily relied on self-report with few using objective measures of PA.^14–16^ The most consistent correlates identified include age, sex and health indicators like physical function, with insufficient evidence for most other factors.^14–16^ Reviews of existing studies have identified a need for more research using representative samples of population-based older people, in addition to more objective assessments of PA.^14–16^ In fact, studies using objective PA measures have identified similar correlates including age, sex and health status.^17,18^ In previous descriptive analyses, we showed that walking speed and self-reported higher impact PA were related to accelerometer-measured higher impact PA among older adults from the general population,^19^ and that older age and worse physical performance were related to lower levels of high-impact PA among older adults attending an aerobics class.^13^ Further, in a recent qualitative study, we showed that older adults identified a fear of falling as a barrier to high-impact activities and that those with joint replacement reported being advised against high-impact PA by their surgeons.^20^ However, no previous study has performed a detailed quantitative analysis of the range of factors associated with accelerometer-measured PA producing rare but highly osteogenic vertical impacts at older age. Further, examining differences in how factors relate to high-impact and overall PA may provide useful insights for intervention design.

The aim of this study was to examine the associations between demographic, behavioural, physiological, psychological and social factors and accelerometer-measured high-impact PA among a population-based sample of older adults. Given that many of these factors are likely to be interrelated, an important secondary aim was to identify which factors were independently associated with high-impact PA in later life. We also examined how these same factors relate to an objective estimate of overall PA.

## Methods

### Study population

Participants were from the Vertical Impacts on Bone in the Elderly (VIBE) study, a multicohort collaboration initially set up to investigate the health consequences of higher impact PA across three population-based cohorts of older people; the Cohort for Skeletal Health in Bristol and Avon (COSHIBA), Hertfordshire Cohort Study (HCS) and the Medical Research Council National Survey of Health and Development (MRC NSHD).^19^ COSHIBA is a representative population-based cohort of 3200 women recruited through fifteen general practices in the Bristol and Avon area during 2007–09.^21^ Only the 1286 COSHIBA participants who consented to be contacted about future research studies in 2014 and who remained resident in the Bristol and Avon area were eligible to participate in the VIBE study. NSHD is a nationally representative sample of 5362 singleton births from one week in March 1946.^22,23^ Most participants (79%) included in the home visit phase of the NSHD 24th data collection (2015–16)^24^ were invited to participate in the VIBE study. HCS comprises 3225 singleton births in Hertfordshire between 1931 and 1939 and who still lived in the area during 1998–2003.^25^ Only the 443 HCS participants who were previously included in the UK arm of the European Project on Osteoarthritis (EPOSA)^26^ were invited to participate in VIBE.

Separate regional ethical approval was obtained for data collection in NSHD (14/LO/1073 and 14/SS/1009), HCS (10/HO311/59) and COSHIBA (14/SW/0138) and written informed consent was obtained from all participants.

### Accelerometer measurements and data processing

Participants who were invited and agreed to accelerometry monitoring, subject to availability of monitors, were provided with a GCDC X15-1c triaxial accelerometer (Gulf Coast Data Concepts, Waveland, Mississippi), custom designed size specific elasticated belt, a time log and a stamped addressed package along with written and, if seen in clinic (COSHIBA) or during a nurse home visit (NSHD), verbal instructions. Accelerometers were configured with standardized settings prior to participant use with a sampling frequency of 50 Hz, a deadband setting of 0.1 g (the threshold which must be exceeded before a recording is made) and a timeout setting of 10 s (a single sample every 10 s is forced even if the recording is <0.1 g). Participants were instructed to wear the accelerometer securely positioned in the belt over their right hip pointing toward the centre of their body for 7 continuous days, removing only for sleeping, washing and swimming. A time log was provided for participants to record when the monitor was put on in the morning and taken off at night for each monitoring day and to state if there was any reason why that day had not been reflective of their normal activity.

Following standardized cleaning and processing (to remove movement artefacts and non-wear time), described in detail elsewhere,^12^ we derived a measure of high-impact PA based on vertical (i.e. *Y*-axis) accelerations peaks (i.e. accelerations higher than the preceding and subsequent readings^12^) measuring ≥1.5 g. The ≥1.5 g cut-point was selected as very few acceleration peaks were observed within higher g bands.^8,12,19^ To examine differences in how each factor relates to high-impact PA and total PA, we derived a measure of overall PA by summing the number of triaxial (i.e. *X*, *Y* and *Z* axes) accelerations peaks measuring ≥0.5 g (i.e. all movements producing both lower and higher magnitude impacts). Periods of inactivity were removed by excluding movements producing ≤0.5 g, and activity data were normalized for wear time based on 7 valid days (≥10 h recording time) of 14 h.^12^ All *g* values represent *g* over and above 1 g from earth’s gravitational force.

### Hypothesized correlates of high-impact PA

The factors hypothesized to be associated with higher impact PA at old age (Table 1) were selected based on previous literature on correlates of PA in older adults.^13–16,19,20,27–29^ These factors were grouped into demographic (age, sex, educational level, occupational class, marital status), behavioural (regular active transport, self-reported time spent in moderate-high-impact PA, smoking and alcohol status), physiological (body mass index (BMI), walking speed, falls, walking restricted due to pain, joint replacement, mobility aid use, difficulty walking (limping), fractures since age 45) and psychological and social domains (self-rated health, fear of falling, mental wellbeing, sleep quality, and contact with relatives, friends and neighbours). A detailed description of each factor including any harmonization process performed for data analysis is provided in Table 1.

**Table 1:** Hypothesized demographic, behavioural, physiological, psychological and social correlates of high-impact physical activity (PA) in older age, COSHIBA, HCS and MRC NSHD, UK, 2015

Domain/factor	Description/question	Categories/units
Demographic factors		
Age	From date of birth	Continuous (per year increase)
Sex	Reported by participants	Female (ref) versus male
Educational level	Highest level by age 26: None; GCE O level; GCE A level; first degree; higher degrees; and other categories. Prospectively reported in NSHD and recalled in COSHIBA and HCS	Three categories: None; up to and including O level, A level and above
Occupational class	Main occupation during working life from participant and their spouse (if married) assigned a 1990 Standard Occupational Classification code	Four categories: Sales, plant and other; craft and personal; associate and clerical; managers and professional
Marital status	COSHIBA and HCS: ‘What is your current marital status? Single and never been married; married and living with husband/wife; married and separated from husband/wife; divorced; widowed; registered/civil partnership; cohabiting’	Married (ref) versus divorced or separated or never married or widowed or other
	NSHD: ‘Are you currently: single, that is never married; married and living with husband/wife; married and separated from husband/wife; divorced; widowed’	
Behavioural factors		
Regular active transport	‘Do you make regular journeys every day or most days by walking, cycling or both? No; yes, i walk; yes, i cycle, yes, i walk and cycle’	No (ref) versus yes (walking and/or cycling)
Self-reported moderate-to-high-impact PA	Hours spent in the last 7 days doing each of aerobics, badminton, dancing, football, hockey, running/jogging, squash, tennis, sprinting	Continuous (*z* score: per increasing time)
Smoking	Participants were asked if they are a current smoker. Information was also collected on previous smoking status	No (includes former smokers) (ref) versus yes
Alcohol	‘Have you drunk alcohol in last year?’	No (ref) versus yes
Physiological factors		
BMI	Weight (kg)/height (m)^2^. Standing heights and weights were measured using standardized protocols in COSHIBA and NSHD^a^: Heights and weights were self-reported in HCS and cross-checked against earlier measured heights and weights	Continuous (*z* score: per higher BMI)
Walking speed	‘Which of the following best describes your walking speed? Unable to walk, very slow, stroll at an easy pace, normal speed, fairly brisk, fast’	Four categories: Unable to walk or very slow; stroll; normal; brisk or fast
Recent falls	COSHIBA and HCS: ‘Have you had any fall, including a slip or trip, in which you lost your balance and landed on the floor or ground or lower level in the past 12 months?’ No; yes, once; yes, twice; yes, three times; yes, four or more times	No (ref) versus yes
	NSHD: ‘In the past 12 months have you had any fall including a slip or trip in which you lost your balance and landed on the floor or ground or lower level?’ No; yes	
Walking restricted due to pain	‘Is your ability to walk restricted due to pain?’	No (ref) versus yes
Joint replacement	‘Have you had a joint replacement?’	No (ref) versus yes
Regular use of mobility aid	Regular use of aids to get around: No; walking stick; zimmer frame; trolley/frame; mobility scooter; wheelchair; other	No (ref) versus yes
Noticeable limp	‘Do you have a noticeable limp?’	No (ref) versus yes
Fractures since age 45	COSHIBA and HCS: ‘Has a doctor told you that you had broken, fractured or chipped any bones since the age of 45? No; yes’	No (ref) versus yes
	NSHD; asked at age 60–64: ‘Have you broken a bone since you were 25 years old?’ Follow-up questions also asked on age, site and cause for each break. These were used to derive a comparable measure of broken bones since age 45	
Psychological and social factors		
Mental wellbeing	Based on responses to the Warwick-Edinburgh Mental Wellbeing Scale^30^	Continuous (*z* score: per higher scores)
Self-rated health	Reported from very good to very poor in HCS and COSHIBA and from excellent to poor in NSHD	Three categories: Very good or excellent (ref); good; fair or poor
Fear of falling	‘Are you worried about falling? No; yes’	No (ref), yes
Sleep	HCS and NSHD: Pittsburgh Sleep Quality Index. COSHIBA: Questions asked about problems trying to get to sleep, including on how many nights/week	Continuous (*z* score from better to worse sleep): HCS and NSHD only
Contact with relatives, friends and neighbours	COSHIBA: ‘How often do you speak to children, siblings, friends and neighbours’ NSHD: How often do you visit/are visited by relatives, friends. HCS: Based on responses to the Lubben Social Network Scale^31^	Analysed separately in each cohort: Speak to each group (COSHIBA) and visit each group (NSHD) ≥once/week (ref) versus <once/week. HCS: Continuous (*z* score)

^a^In COSHIBA, height was measured to the nearest mm using a Harpenden stadiometer and weight to the nearest 50 g using Tanita weighing scales whereas in NSHD height was measured to the nearest mm using a Leicester stadiometer and weight to the nearest 100 g using Tanita weighing scales. Ref: reference category.

### Statistical analyses

Means and standard deviations were used to summarize continuous measures and proportions to describe categorical measures. Differences in each measure between the three participating cohorts were investigated using ANOVA for continuous measures and chi-squared tests for categorical measures and where only two cohorts had relevant data, differences were examined using *t*-tests for continuous measures and Chi-squared tests for categorical measures. Accelerometer data were expressed as medians and interquartile ranges due to their skewed distributions and, differences between cohorts were examined using a nonparametric *k*-sample equality of medians test. Linear regression was subsequently used to examine associations between each selected factor and high-impact PA, and overall PA. Interaction terms were used to test cohort differences in associations of each factor with accelerometer outcomes and subsequent analyses were performed on all participants combined with cohort-adjustment after little evidence of interactions was found. Interaction terms were also used to test sex-differences and subsequently men and women were combined with adjustment made for sex after no evidence of sex-interaction was found. For categorical factors, deviation from linearity was tested by comparing models with categorical exposures to models with same exposure entered as a continuous term, and where no evidence of deviation from linearity was found they were treated as continuous terms (with estimates representing a per category change).

First models were adjusted for age, sex and cohort. Second models included adjustment for all factors within each domain followed by final third models that were concurrently adjusted for all factors from all domains. Included in these second and third models were all those factors with a statistical significance of *P* ≤ 0.1 from tests of association with higher impact PA and/or overall PA in the first (age, sex and cohort-adjusted) models. Accelerometer outcomes were log-transformed due to their skewed distributions. To minimize the potential for bias due to missing data, we used multiple imputation by chained equations^32^ to impute missing data for each factor thereby including all participants with valid accelerometer outcomes. Imputation models were run using 20 multiply imputed datasets which were combined using Rubin’s combination rules. Results from imputed datasets were similar to results from complete case analysis and the former are presented. We also calculated adjusted *R*^2^ of our final models to identify how much variance in high-impact PA and overall PA was explained by the selected factors, after accounting for important predictors and those which were not independently related to outcomes in final model.

## Results

### Descriptive statistics

A total of 1187 participants aged between 69 and 88 years (mean = 72.4) (72.8% females) had valid measures of PA from accelerometers. Of these, 430 were from COSHIBA (100% females, mean age = 76.6), 649 from NSHD (50.2% females, mean age = 69) and 108 from HCS (42.1% females, mean age = 78.4). Men had greater levels of high-impact PA and overall PA than women (Table 2). Greater levels of both high-impact PA and overall PA were recorded in NSHD than in COSHIBA or HCS, reflecting their younger age and a higher proportion of males (Table 2). High-impact PA and overall PA were moderately correlated (Pearson correlation coefficient between log-high-impact PA and log-overall PA = 0.6). Distribution of each selected factor by cohort is provided in Table 2.

**Table 2:** Participant characteristics.

	COSHIBA (*n* = 430)	HCS (*n* = 108)	MRC NSHD (*n* = 649)	*P* for cohort difference
Accelerometer wear time	5.2 (1.8)	4.2 (2.2)	4.9 (2.1)	<0.001
Counts of high magnitude (≥1.5 g) vertical acceleration peaks (high-impact PA)*	42 (18, 106)	40 (13, 122)	93 (35, 271)	<0.001
Counts of low and high magnitude (≥ 0.5 g) triaxial acceleration peaks (overall PA)*	19 743 (11 156, 32 290)	21 516 (11 628, 32 081)	36 329 (22 834, 53 100)	<0.001
Demographic factors				
Age	76.8 (3.0)	78.5 (2.6)	69(0)	<0.001
Female	430 (100)	43 (39.8)	317 (48.8)	<0.001
Educational level by age 26				<0.001
None	175 (42.6)	62 (60.2)	170 (27.3)	
Up to and including O level	138 (33.6)	28 (27.2)	169 (27.2)	
A level and above	98 (23.8)	13 (12.6)	283 (45.5)	
Highest 1990 Standard Occupational Classification (SOC90)				<0.001
1–2 (highest)	200 (49.8)	36 (35.0)	333 (54.2)	
3–4	128 (31.8)	24 (23.3)	170 (27.6)	
5–6	55 (13.7)	24 (23.3)	83 (13.5)	
7–8 (lowest)	19 (4.7)	19 (18.5)	29 (4.7)	
Married	208 (48.7)	74 (69.8)	484 (79.5)	<0.001
Behavioural factors				
Regular walking and/or cycling	282 (66.7)	81 (75.0)	431 (67.8)	0.2
Self-reported higher impact PA	0.6 (1.2)	0.3 (1.0)	0.3 (0.9)	<0.001
Current smoker	19 (4.5)	3 (2.8)	36 (5.6)	0.4
Drunk alcohol in last year	346 (81.0)	94 (87.0)	569 (93.4)	<0.001
Physiological factors				
BMI (kg/m^2^)	27.1 (4.7)	25.6 (3.4)	27.3 (4.2)	0.002
Walking speed				<0.001
Unable to walk or very slow	34 (7.9)	10 (9.3)	15 (2.3)	
Stroll at an easy pace	100 (23.4)	36 (33.3)	89 (13.8)	
Normal speed	191 (44.6)	47 (43.5)	338 (52.6)	
Fairly brisk or fast	103 (24.1)	15 (13.9)	201 (31.3)	
Walking restricted by pain	144 (35.2)	39 (36.1)	123 (19.2)	<0.001
Regularly uses mobility aid	62 (20.1)	15 (17.9)	21 (3.30)	<0.001
Has noticeable limp	51 (12.1)	19 (17.9)	44 (7.0)	<0.001
Had joint replacement	85 (20.0)	23 (21.3)	43 (6.7)	<0.001
Had fall in last year	135 (32.1)	25 (23.6)	121 (19.7)	<0.001
Fractures since age 45	152 (36.3)	23 (21.7)	134 (26.2)	0.001
Psychological and social factors				
WEMWBS score	54.7 (8.9)	52.6 (8.5)	53.6 (8.3)	0.02
Self-rated health				<0.001
Very good or excellent	106 (24.9)	16 (15.4)	355 (57.7)	
Good	237 (55.8)	60 (57.7)	191 (31.1)	
Fair or poor/very poor	82 (19.3)	28 (26.9)	69 (11.2)	
Fear of falling	36 (8.5)	6 (5.6)	9 (1.4)	<0.001
Pittsburgh Sleep Quality Index	–	5.2 (3.4)	4.9 (3.2)	0.2
Speak with children ≥ once/week	370 (96.1)		–	
Speak with siblings ≥ once/week	194 (63.8)		–	
Speak with friends ≥ once/week	382 (93.2)		–	
Speak with neighbours ≥ once/week	366 (89.9)		–	
Visit or visited by relatives ≥ once/week	–	–	360 (58.5)	
Visit or visited by friends ≥ once/week	–	–	356 (57.9)	
Lubben Social Network Scale score	–	17.9 (5.1)	–	

Data in table show number (%) for categorical measures and mean (standard deviation) for continuous measures. *This data shows median and interquartile range. Sample size varies depending on those with data on each characteristic. SD, standard deviation.

### Correlates of high-impact PA

In minimally adjusted models (Table 3), older age, female sex, lower education level and occupational class, lack of regular active transport, less self-reported time spent in higher impact PA, current smokers, higher BMI, slower walking speed, experiencing pain during walking, regular use of a mobility aid, presence of a noticeable limp, lower mental wellbeing, poorer self-rated health and a fear of falling were all associated with lower levels of high-impact PA. Conversely, marital status, alcohol drinking, recent falls, previous fracture and sleep quality were unrelated to high-impact PA whereas the association of previous joint replacement with high-impact PA was borderline (Table 3). Similarly, speaking with friends and relatives (COSHIBA), visiting or being visited by friends and relatives (NSHD) and the Lubben Social Network Scale (HCS) were not associated with high-impact PA (not shown).

**Table 3:** Correlates of high-impact PA and overall PA among older adults. Estimated from minimally adjusted models (*n* = 1187).

	Log-high-impact PA		Log-overall PA	
	*β* (95% CI)	*P*	*β* (95% CI)	*P*
Demographic factors				
Age (per year increase)	−0.09 (−0.13, −0.05)	<0.001	−0.07 (−0.09, −0.05)	<0.001
Sex (male)	0.42 (0.22, 0.63)	<0.001	0.22 (0.12, 0.32)	<0.001
Educational level	0.27 (0.16, 0.37)	<0.001	0.14 (0.09, 0.19)	<0.001
Occupational class	0.17 (0.08, 0.27)	<0.001	0.06 (0.01, 0.11)	0.02
Marital Status (married)	−0.10 (−0.29, 0.09)	0.3	0.00 (−0.09, 0.10)	>0.9
Behavioural factors				
Regular walking and/or cycling (yes)	0.43 (0.26, 0.61)	<0.001	0.57 (0.48, 0.65)	<0.001
SR higher impact PA (per SD increase)	0.29 (0.21, 0.37)	<0.001	0.11 (0.07, 0.15)	<0.001
Smoking status (current smoker)	−0.49 (−0.87, −0.10)	0.01	−0.31 (−0.50, −0.12)	0.002
Drank alcohol in last year (yes)	0.10 (−0.16, 0.36)	0.5	0.19 (0.06, 0.32)	0.005
Physiological factors				
BMI (per SD increase)	−0.24 (−0.33, −0.16)	<0.001	−0.26 (−0.30, −0.22)	<0.001
Walking speed	0.41 (0.33, 0.50)	<0.001	0.38 (0.34, 0.42)	<0.001
Walking restricted due to pain	−0.49 (−0.68, −0.30)	<0.001	−0.49 (−0.58, −0.40)	<0.001
Regularly uses mobility aid	−0.76 (−1.06, −0.46)	<0.001	−0.79 (−0.94, −0.65)	<0.001
Noticeable limp (yes)	−0.37 (−0.65, −0.08)	0.01	−0.46 (−0.59, −0.32)	<0.001
Joint replacement (yes)	−0.20 (−0.46, 0.06)	0.1	−0.25 (−0.37, −0.12)	<0.001
Fall in last year (yes)	0.01 (−0.19, 0.21)	0.9	−0.16 (−0.26, −0.06)	0.001
Fracture since age 45 (yes)	0.01 (−0.19, 0.21)	0.9	0.02 (−0.08, 0.12)	0.7
Psychological and social factors				
WEMWBS (per SD increase)	0.11 (0.02, 0.19)	0.02	0.08 (0.04, 0.12)	<0.001
Self-rated health		<0.001		<0.001
Very good or excellent	1.00		1.00	
Good	−0.57 (−0.76, −0.38)		−0.28 (−0.37, −0.19)	
Fair or poor/very poor	−1.00 (−1.25, −0.75)		−0.70 (−0.82, −0.58)	
Fear of falling (yes)	−0.91 (−1.33, −0.49)	<0.001	−0.90 (−1.10, −0.70)	<0.001
PSQI (per SD increase) (HCS and NSHD)	−0.04 (−0.17, 0.08)	0.5	−0.07 (−0.13, −0.02)	0.009

Adjusted for age, sex and cohort. High-impact PA defined as vertical (*Y*-axis) accelerations peaks ≥1.5 g. Overall PA defined as triaxial accelerations peaks ≥0.5 g. Educational level: per category unit change from lower to higher. Occupational class: per category unit change from lower to higher. Walking speed: per category unit change from slower to faster. SD, standard deviation; WEMWBS, Warwick-Edinburgh Mental Wellbeing Scale (higher scores represent better wellbeing); PSQI, Pittsburgh Sleep Quality Index (higher scores represent worse sleep quality).

Following mutual adjustment for all factors showing initial associations, age, sex, education, active transport, self-reported high-impact PA, walking speed and self-rated health were all independently related to high-impact PA whereas occupational class, BMI, recent falls and sleep quality showed borderline associations with high-impact PA (Table 4). On the other hand, smoking status, pain during walking, mobility aid use, noticeable limp, mental wellbeing and fear of falling were no longer related to high-impact PA after adjustment (Table 4). The demographic, behavioural, physiological and psychological/social factors explained 12% (adjusted *R*^2^ = 0.119, min = 0.117, max = 0.124); 14.8% (adjusted *R*^2^ = 0.148, min = 0.147, max = 0.15); 16.6% (adjusted *R*^2^ = 0.166, min = 0.164, max = 0.168); and 15.2% (adjusted *R*^2^ = 0.152, min = 0.148, max = 0.156) of between-participant variance in high-impact PA respectively. The final model explained 23% of between-participant variance in high-impact PA (adjusted *R*^2^ = 0.232, min = 0.226, max = 0.242).

**Table 4:** Correlates of high-impact PA and overall PA among older adults. Estimated from domain-specific and fully adjusted models (*n* = 1187).

	Log-high-impact PA				Log-overall PA			
	Domain-specific models, *β* (95% CI)	*P*	Fully adjusted models, *β* (95% CI)	*P*	Domain-specific models, *β* (95% CI)	*P*	Fully adjusted models, *β* (95% CI)	*P*
Demographic factors								
Age (per year increase)	−0.09 (−0.13, −0.05)	<0.001	−0.05 (−0.09, −0.01)	0.03	−0.07 (−0.09, −0.05)	<0.001	−0.04 (−0.05, −0.02)	<0.001
Sex (male)	0.43 (0.22, 0.64)	<0.001	0.50 (0.29, 0.71)	<0.001	0.22 (0.11, 0.32)	<0.001	0.20 (0.11, 0.29)	<0.001
Educational level	0.23 (0.11, 0.34)	<0.001	0.15 (0.04, 0.26)	0.008	0.13 (0.08, 0.19)	<0.001	0.08 (0.04, 0.13)	<0.001
Occupational class	0.10 (0.00, 0.21)	0.05	0.07 (−0.03, 0.17)	0.2	0.02 (−0.03, 0.07)	0.5	−0.02 (−0.06, 0.02)	0.4
Behavioural factors								
Regular walking and/or cycling (yes)	0.38 (0.20, 0.55)	<0.001	0.22 (0.04, 0.40)	0.02	0.55 (0.46, 0.63)	<0.001	0.37 (0.30, 0.45)	<0.001
SR higher impact PA (per SD increase)	0.27 (0.19, 0.35)	<0.001	0.22 (0.14, 0.30)	<0.001	0.08 (0.04, 0.12)	<0.001	0.03 (−0.01, 0.06)	0.1
Smoking status (current smoker)	−0.44 (−0.82, −0.07)	0.02	−0.21 (−0.59, 0.16)	0.3	−0.26 (−0.43, −0.08)	0.004	−0.16 (−0.32, −0.01)	0.04
Drank alcohol in last year (yes)	0.05 (−0.21, 0.31)	0.7	−0.18 (−0.44, 0.08)	0.2	0.17 (0.05, 0.29)	0.006	0.02 (−0.09, 0.13)	0.7
Physiological factors								
BMI (per SD increase)	−0.13 (−0.22, −0.04)	0.003	−0.08 (−0.17, 0.00)	0.05	−0.15 (−0.19, −0.11)	<0.001	−0.13 (−0.16, 0.09)	<0.001
Walking speed	0.34 (0.23, 0.45)	<0.001	0.23 (0.11, 0.34)	<0.001	0.28 (0.23, 0.32)	<0.001	0.21 (0.16, 0.26)	<0.001
Walking restricted due to pain	−0.13 (−0.35, 0.09)	0.2	−0.01 (−0.24, 0.21)	0.9	−0.12 (−0.22, −0.03)	0.01	−0.04 (−0.14, 0.05)	0.4
Regularly uses mobility aid	−0.24 (−0.61, 0.14)	0.2	−0.17 (−0.56, 0.21)	0.4	−0.26 (−0.42, −0.10)	0.002	−0.26 (−0.43, −0.10)	0.002
Noticeable limp (yes)	0.24 (−0.09, 0.57)	0.2	0.23 (−0.09, 0.56)	0.2	0.12 (−0.02, 0.26)	0.1	0.09 (−0.05, 0.22)	0.2
Joint replacement (yes)	−0.04 (−0.29, 0.22)	0.8	−0.01 (−0.26, 0.24)	>0.9	−0.02 (−0.10, 0.07)	0.7	−0.05 (−0.15, 0.06)	0.4
Fall in last year (yes)	0.16 (−0.04, 0.36)	0.1	0.14 (−0.05, 0.34)	0.1			−0.02 (−0.10, 0.06)	0.6
Psychological and social factors								
WEMWBS (per SD increase)	−0.01 (−0.10, 0.09)	0.9	0.01 (−0.09, 0.10)	0.9	−0.01 (−0.06, 0.03)	0.6	−0.01 (−0.05, 0.03)	0.6
Self-rated health		<0.001		<0.001		<0.001		0.002
Very good or excellent	1.00		1.00		1.00		1.00	
Good	−0.62 (−0.81, −0.42)		−0.43 (−0.63, −0.23)		−0.29 (−0.39, −0.20)		−0.14 (−0.22, −0.05)	
Fair or poor/very poor	−1.00 (−1.28, −0.71)		−0.56 (−0.86, −0.25)		−0.60 (−0.74, −0.47)		−0.19 (−0.32, −0.07)	
Fear of falling (yes)	−0.63 (−1.07, −0.19)	0.005	−0.17 (−0.66, 0.33)	0.5	−0.66 (−0.87, −0.45)	<0.001	−0.03 (−0.24, 0.18)	0.8
PSQI (per SD increase) (HCS and NSHD)	0.10 (−0.03, 0.24)	0.1	0.11 (−0.02, 0.25)	0.09	0.00 (−0.06, 0.06)	0.9	0.01 (−0.04, 0.06)	0.8

Domain-specific models: adjusted for age, sex, cohort and all factors from the same domain. Fully adjusted models: adjusted for age, sex, cohort and all factors in table. High-impact PA defined as vertical (*Y*-axis) acceleration peaks ≥1.5 g. Overall PA defined as triaxial acceleration peaks ≥0.5 g. Educational level: per category unit change from lower to higher. Occupational class: per category unit change from lower to higher. Walking speed: per category unit change from slower to faster. SD, standard deviation. WEMWBS, Warwick-Edinburgh Mental Wellbeing Scale (higher scores represent better wellbeing). PSQI, Pittsburgh Sleep Quality Index (higher scores represent worse sleep quality).

### Correlates of overall PA

Except for marital status and previous fracture, all other factors examined were associated with overall PA in models with minimum adjustments (Table 3). As with high-impact PA, the social network measures were also unrelated to overall PA (not shown) though a weak association was observed in NSHD between regularly visiting friends and higher overall PA (sex-adjusted difference in log-overall PA for visiting friends less than once/week versus at least once/week was −0.11 (95% confidence intervals: −0.22, 0.00, *P* = 0.05).

After mutual adjustment for all factors showing initial associations with PA outcomes, age, sex, education, active transport, walking speed and self-rated health all independently predicted overall PA (Table 4). In addition, and contrary to findings for high-impact PA, smoking status, BMI and mobility aid use were also independently associated with overall PA whereas self-reported high-impact PA and sleep quality showed little evidence of associations with overall PA (Table 4). The demographic, behavioural, physiological, and psychological/social factors explained 20.7% (adjusted *R*^2^ = 0.207, min = 0.204, max = 0.211), 31.6% (adjusted *R*^2^ = 0.316, min = 0.312, max = 0.32), 42.5% (adjusted *R*^2^ = 0.425, min = 0.422, max = 0.429) and 29.3% (adjusted *R*^2^ = 0.293, min = 0.288, max = 0.298) of between-participant variance in overall PA, respectively. The final model explained 48% of between-participant variance in overall PA (adjusted *R*^2^ = 0.482, min = 0.477, max = 0.486).

## Discussion

### Main finding of this study

We examined the associations of a wide range of demographic, behavioural, physiological, psychological and social factors with accelerometer-measured high-impact PA among participants aged in their late 60s, 70s and 80s recruited from three British population-based cohorts. Besides an older age and female sex, several factors, namely, lower education, lack of regular active transport, slower walking speed, less reported time in high-impact PA and poorer self-rated health were all independently associated with lower levels of high-impact PA in later life. On the other hand, smoking status, pain during walking, mobility aid use, noticeable limp, mental wellbeing and fear of falling were only related to high-impact PA prior to adjustment for other factors. All factors combined explained nearly a quarter of the variance in levels of high-impact PA between individuals. Moreover, while broadly similar findings were observed when examining correlates of overall PA (i.e. PA encompassing lower as well as higher impacts), there were important differences. Specifically, BMI, smoking status and mobility aid use were independently associated with overall but not high-impact PA whereas, based on qualitative assessment of differences in effect size, reported higher impact PA, sleep quality, education and self-rated health appeared more strongly related to high-impact PA. Walking speed was an important correlate of both high-impact PA and overall PA.

### What is already known on this topic

This is the first study to examine the correlates of high-impact PA assessed by accelerometer at old age however, some of the factors identified as predictive of higher impact PA are similar to factors related to overall PA in previous studies of older adults. For example, regular PA reported by 8881 Australians aged 65+ years was independently associated with male sex, younger age, ability to travel independently, better physical functioning and lower psychological distress whereas no independent associations were found for employment status or fear of falling. Likewise, among a large sample of 48–83-year-old Swedish women, reported PA was lower with increasing age, BMI and in smokers.^29^ PA counts per minute assessed via accelerometers in 850 70–77-year-old Norwegians were related to cardiorespiratory fitness and sex but not social support,^17^ while among 560 British adults aged at least 65 years, independent predictors of average daily accelerometer step-counts included age, general health, disability, BMI and number of long walks.^18^

### What this study adds

Our findings are important as they offer a first look at factors related to accelerometer-measured higher impact PA which is thought to be important for bone health in older populations. Consistent with and extending our previous unadjusted analyses^19^ is that walking speed, a strong predictor of survival,^33^ was related to both high-impact and overall PA even after adjustment, which may suggest an important role for underlying physical function. Likewise, self-rated health predicted both high-impact and overall PA, but appeared more strongly related to high-impact PA, which may reflect effects of underlying physical health.^34^ In addition, reported high-impact PA was strongly predictive of accelerometer-measured high-impact PA, but not overall PA, including after adjustment, which indicates that our objective measure of high-impact PA is capturing time spent in high-impact activities.

That education was related to both high-impact and overall PA is consistent with studies showing lower self-reported and objectively measured PA among older adults from lower socioeconomic backgrounds,^35^ as well as with our qualitative findings that older adults identified greater knowledge of exercise benefits to be a facilitator of higher impact PA.^20^ Fear of falling was initially related to high-impact PA, as previous reported in our qualitative study,^20^ however, that this association, and that with other markers of functional status, was lost after adjustment may be because it was captured by other model covariates like self-rated health. Finally, that regular active transport was related to higher counts of both PA parameters might reflect effects of active lifestyles.^36^

### Limitations of this study

Strengths of this study include use of raw accelerometer recordings to derive objective measures of high-impact PA, comparison of findings with an objective measure of overall PA and inclusion of three population-based cohorts encompassing a broad age range of older individuals helps to increase power and generalizability of findings. Limitations of this study include its cross-sectional study design, which precludes inference regarding causality especially as reverse causation is possible. Allocation of the independent variables into domains was subjective which is another potential limitation of our approach, however this allowed for an organized sequential analysis. Additionally, as many correlated variables were simultaneously adjusted for in our final models these may be over-adjusted leading to an underestimation in effect sizes. It was also not possible to examine levels of PA based on conventional measures of energy expenditure as the GCDC accelerometers only recorded PA impact magnitude. Furthermore, we only had information on individual-level factors in VIBE, however, both perceived and observed environmental characteristics have been associated with PA^37,38^ and thus future studies should investigate how they might relate to high-impact PA. Of further consideration is that VIBE participants tended to have lower BMI and higher educational level compared with others who did not participate in VIBE^19^ and this selection bias may have led to underestimations of associations. Finally, measurement error in some of the factors studied might influence our findings, as could residual confounding due to unmeasured factors.

### Implications and conclusions

Our findings suggest that maintaining physical function, wellbeing and health may be important for promoting osteogenic PA in later life. Further, certain groups of older people such as those with lower educational qualifications may benefit from supportive interventions to increase higher impact PA, whereas older women of any age and the oldest men and women may both be target populations for interventions. In conclusion, by using accelerometers calibrated to detect high magnitude vertical impacts from ground reaction forces, we showed that several factors were independently associated with osteogenic PA in older British men and women.
